# Human Cachexia Induces Changes in Mitochondria, Autophagy and Apoptosis in the Skeletal Muscle

**DOI:** 10.3390/cancers11091264

**Published:** 2019-08-28

**Authors:** Gabriela S. de Castro, Estefania Simoes, Joanna D.C.C. Lima, Milene Ortiz-Silva, William T. Festuccia, Flávio Tokeshi, Paulo S. Alcântara, José P. Otoch, Dario Coletti, Marilia Seelaender

**Affiliations:** 1 Cancer Metabolism Research Group, Department of Cell and Tissue Biology, Institute of Biomedical Sciences, University of São Paulo, 05508-900 São Paulo, Brazil; 2 Department of Physiology & Biophysics, Institute of Biomedical Sciences, University of São Paulo, 05508-900 São Paulo, Brazil; 3 Department of Clinical Surgery, Faculty of Medicine, University of São Paulo, 01246-903 São Paulo, Brazil; 4 Department of Biological Adaptation and Aging, B2A (CNRS UMR 8256-INSERM ERL U1164-UPMC P6), Sorbonne University, 75005 Paris, France

**Keywords:** cancer cachexia, skeletal muscle, mitochondria, autophagy, apoptosis

## Abstract

Cachexia is a wasting syndrome characterized by the continuous loss of skeletal muscle mass due to imbalance between protein synthesis and degradation, which is related with poor prognosis and compromised quality of life. Dysfunctional mitochondria are associated with lower muscle strength and muscle atrophy in cancer patients, yet poorly described in human cachexia. We herein investigated mitochondrial morphology, autophagy and apoptosis in the skeletal muscle of patients with gastrointestinal cancer-associated cachexia (CC), as compared with a weight-stable cancer group (WSC). CC showed prominent weight loss and increased circulating levels of serum C-reactive protein, lower body mass index and decreased circulating hemoglobin, when compared to WSC. Electron microscopy analysis revealed an increase in intermyofibrillar mitochondrial area in CC, as compared to WSC. Relative gene expression of Fission 1, a protein related to mitochondrial fission, was increased in CC, as compared to WSC. LC3 II, autophagy-related (ATG) 5 and 7 essential proteins for autophagosome formation, presented higher content in the cachectic group. Protein levels of phosphorylated p53 (Ser46), activated caspase 8 (Asp384) and 9 (Asp315) were also increased in the skeletal muscle of CC. Overall, our results demonstrate that human cancer-associated cachexia leads to exacerbated muscle-stress response that may culminate in muscle loss, which is in part due to disruption of mitochondrial morphology, dysfunctional autophagy and increased apoptosis. To the best of our knowledge, this is the first report showing quantitative morphological alterations in skeletal muscle mitochondria in cachectic patients.

## 1. Introduction

Cachexia is a wasting syndrome defined as the continuous loss of skeletal muscle mass due to an imbalance between protein synthesis and degradation, with or without body fat loss that can be partially, but not totally reversed by nutritional support [1,2]. Weight loss is the most prominent characteristic of cachexia and is strongly associated with mortality [1]. Cachexia is highly prevalent in cancer subjects reaching up to 80% of those with advanced disease [3] and is the direct cause of death in at least 20% of all cancer patients [3].

Numerous inflammatory cytokines are involved in the etiology of cachexia, including tumor necrosis factor alpha, interleukin (IL)-6, IL-1 and interferon gamma [3,4] indicating that systemic inflammation plays a central role in cancer-associated cachexia. Noteworthy, the adipose tissue undergoes important tissue remodeling and displays increased lipolysis and exacerbated secretion of inflammatory cytokines, majorly contributing to the systemic inflammation described in cachectic patients [5,6,7,8].

Systemic inflammation is one of the major underlying mechanisms driving the reduction in skeletal muscle mass, which responds for approximately 30% of changes in body weight loss in patients with cachexia [9,10]. In rodents, impairment of mitochondria quality control and function has been reported [10,11,12,13,14] to be precocious, and to trigger events occurring before muscle atrophy in cachexia-related muscle atrophy [15]. Indeed, disruption of skeletal muscle mitochondrial oxidative capacity [16], diminished cytochrome C oxidase and ATP synthase protein content, decreased mitochondrial biogenesis capability and impaired mitochondrial protein quality control were described in rodents with cancer-associated cachexia [12]. Mitochondrial membrane may undergo phospholipid remodeling [13], which could result in changes in mitochondrial fluidity, thus leading to higher susceptibility to apoptosis in the skeletal muscle fiber in patients with cancer-associated cachexia [17]. Furthermore, mitochondrion morphological alterations, such as electron–lucent areas and swelling, have also been reported in models of cancer cachexia [10,14].

Loss of skeletal muscle is an independent predictor of immobility and mortality of cancer patients [18,19]. Muscle depletion is the consequence of an imbalanced protein synthesis and degradation and these processes seem to be dysregulated in patients with cancer cachexia [20]. In animal models of cachexia, the enhanced activity of the ubiquitin-proteasome pathway may drive muscle wasting [3,10,21]. In humans, activation of this system is not a consistent finding, suggesting the activation of other pathways, such as macroautophagy (from now on referred to as autophagy) [22] and apoptosis [17]. Autophagy is a physiological process in which disrupted or malfunctioning organelles, proteins and portions of the cytoplasm are engulfed by an autophagosome vesicle that eventually fuses with the lysosomes for content degradation. Excessive or insufficient autophagy may be both detrimental to muscle fiber function, and a fine balance is necessary in order to preserve muscle mass [23]. Deregulated autophagic flux [22] and excessive autophagy [24] were reported in patients with cancer-associated cachexia. Moreover, apoptosis in the skeletal muscle leads to muscle fiber atrophy and plays an important role in tissue remodeling in muscular dystrophy [25], denervation [26], and as well has been described in cancer-associated muscle wasting in patients and in animal models [17,27,28]. Based on these premises, we investigated the morphology of intermyofibrillar mitochondria and aimed at evaluating the possible activation of autophagy and apoptosis in the skeletal muscle of patients with gastrointestinal cancer-associated cachexia, as compared with patients with weight-stable gastrointestinal tumors. We found that skeletal muscle mitochondria show increased volume in cachectic patients without a decrease in mitochondrial DNA content. Moreover, skeletal muscle autophagy and apoptosis were activated in human cancer-associated cachexia.

## 2. Results

### 2.1. General Parameters

Patients from WSC and CC groups presented similar age, height and body weight at diagnosis, as shown in Table 1. CC presented a median of 15% body weight loss in the last 6 months (*p* < 0.0001) and lower body mass index (BMI) at diagnosis, compared to WSC (*p* = 0.003). Furthermore, CC showed higher serum C-reactive protein (*p* = 0.01) and lower hemoglobin content (*p* < 0.0001), compared to WSC. Although serum albumin was not different between the groups (*p* = 0.28), CRP/albumin ratio was higher in CC patients (*p* = 0.009). Therefore, we chose to adopt this ratio as a more reliable tool for diagnostic purpose [29]. No differences were observed for serum glucose (*p* = 0.67), triacylglycerol (*p* = 0.60), total cholesterol (*p* = 0.055), LDL (*p* = 0.11) and HDL cholesterol (*p* = 0.26).

### 2.2. Mitochondrial Alterations

**Figure 1:**
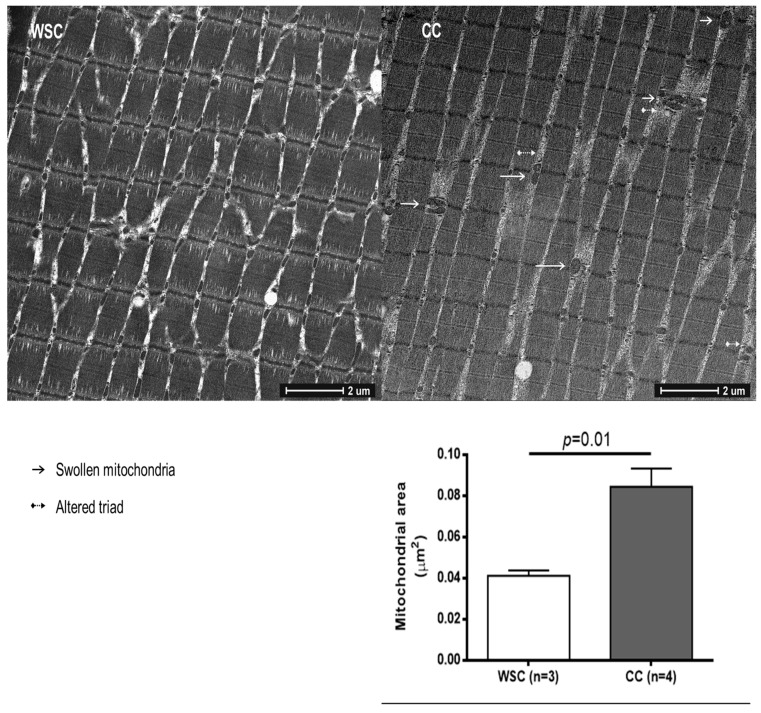
Skeletal muscle photomicrograph and intermyofibrillar mitochondrial area of WSC and CC. Image J software was used to assess mitochondrial area (WSC, *n* = 3; CC, *n* = 4). Mitochondrial area was compared using Student’s *t* test and data are expressed as mean and standard error. WSC—weight-stable cancer patients; CC—cachectic cancer patients.

Images from the transmission electron microscopy showed modifications in the skeletal muscle of CC, characterized by altered mitochondria morphology and disrupted triads—two sarcoplasmic reticulum cisternae associated with T tubules—as illustrated in Figure 1 and Figure S1.

The intermyofibrillar mitochondrial area was larger (*p* = 0.01, Figure 1), and the expression of Fis1 (*p* = 0.03, Table 2) was higher in CC, when compared to WSC. No changes were observed in the gene expression of MFN2 (*p* = 0.20), TFAM (*p* = 0.38) and PGC-1α (*p* = 0.34).

**Figure 2:**
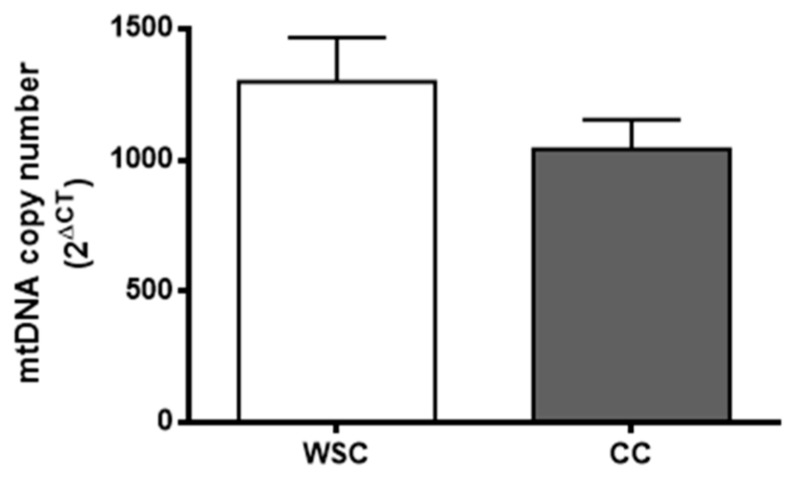
Mitochondrial DNA copy number of WSC and CC subjects. Total DNA was amplified using RT-PCR to assess nuclear DNA and mtDNA copy number (WSC, *n* = 12; CC, *n* = 13). Data are expressed as mean and standard error and Student’s *t* test was used to compare WSC and CC groups. WSC—weight-stable cancer patients; CC—cachectic cancer patients.

Skeletal muscle mitochondrial DNA (mtDNA) copy number did not differ between the groups (*p* = 0.21), as shown in Figure 2.

### 2.3. Autophagy and Apoptosis

**Figure 3:**
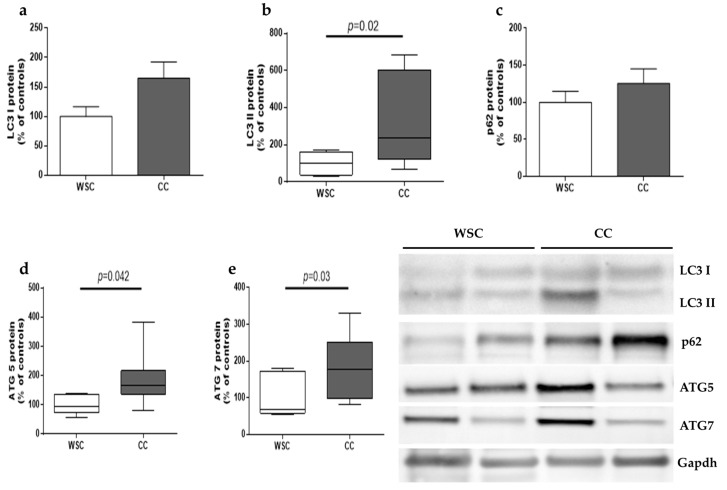
Autophagy-related proteins in skeletal muscle of WSC and CC patients. Western blot analysis of (**a**) LCB3 I (WSC, *n* = 8; CC, *n* = 10); (**b**) LC3B II (WSC, *n* = 8; CC, *n* = 10); (**c**) p62 (WSC, *n* = 8; CC, *n* = 10); (**d**) ATG5 (WSC, *n* = 6; CC, *n* = 8) and (**e**) ATG7 (WSC, *n* = 6; CC, *n* = 8). Data are expressed as mean and standard error and were compared using Student’s *t* test or were represented in box plots and compared using Mann–Whitney test. WSC—weight-stable cancer patients; CC—cachectic cancer patients.

Since dysfunctional mitochondria and other organelles are removed by autophagy, the expression of proteins involved in the process was evaluated. LC3B is a protein crucial for autophagosome formation. LC3B presents two isoforms: LC3BI which is located in the cytosol, can be lipidated to form the second isoform, and LC3B II, which is conjugated with phosphatidylethanolamine and associated to the membrane of the autophagosome. LC3B II protein content was higher in CC compared to WSC (*p* = 0.02), while no significant difference was observed for LC3B I (*p* = 0.08), as illustrated in Figure 3. Another important protein involved in autophagy is p62, which is related to the autophagic flux, given that it binds to ubiquitinated proteins and to LC3B, at the same time, tagging proteins aggregates to be encompassed by autophagosomes. No changes were observed for p62 (*p* = 0.33). ATG5 and ATG7 were upregulated in the skeletal muscle of CC, in relation to WSC (*p* = 0.042 and *p* = 0.03, respectively).

**Figure 4:**
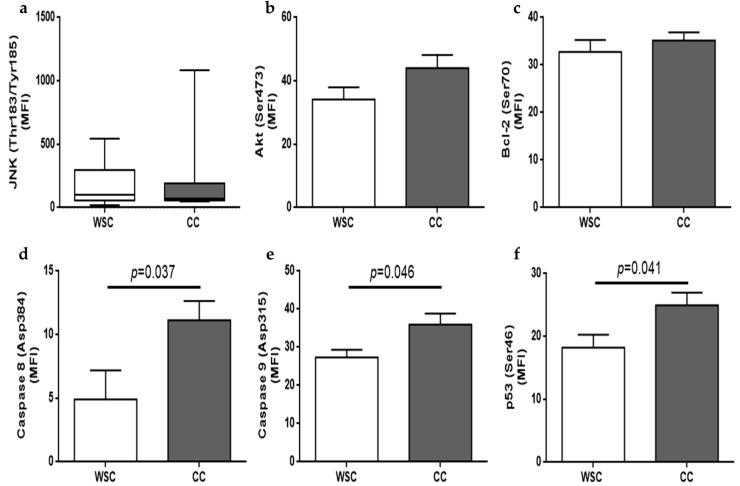
Apoptosis-related protein in skeletal muscle of WSC and CC patients. Multiplex-based analysis of phosphorylated (**a**) JNK (WSC, *n* = 9; CC, *n* = 12); (**b**) phosphorylated Akt (WSC, *n* = 8; CC, *n* = 12); (**c**) phosphorylated Bcl-2 (WSC, *n* = 7; CC, *n* = 11); (**d**) activated caspase 8 (WSC, *n* = 5; CC, *n* = 11); (**e**) activated caspase 9 (WSC, *n* = 7; CC, *n* = 11) and (**f**) phosphorylated p53 (WSC, *n* = 7; CC, *n* = 11). Data are expressed as mean and standard error and were compared using Student’s *t* test or were represented in box plots and compared using Mann–Whitney test. WSC—weight-stable cancer patients; CC—cachectic cancer patients.

Multiplex-based technology was employed to compare apoptosis-related proteins in the skeletal muscle of WSC and CC patients. BAD protein was not detectable and no differences were observed for phosphorylated Akt (*p* = 0.12), phosphorylated Bcl-2 (*p* = 0.52) and phosphorylated JNK (*p* = 0.9). Phosphorylated p53 (Ser46) was increased in CC, as compared to WSC (*p* = 0.041), as well as active caspases 8 (Asp384) (*p* = 0.037) and 9 (Asp315) (*p* = 0.046). Apoptosis-related protein content is shown in Figure 4.

Skeletal muscle apoptosis is also characterized by nuclei morphological alterations, such as presence of apoptotic nuclei, chromatin condensation and marginalization. Following the observation of increased apoptotic markers in the skeletal muscle of CC patients, we examined the electron microscopy samples searching for apoptosis-related morphological alterations [30], such as chromatin condensation and marginalization and apoptotic nuclei showed in Figure S2. These alterations were observed in the skeletal muscle samples of the CC patients. No apoptotic bodies were observed in the WSC patients.

## 3. Discussion

Cancer-associated cachexia may be driven by inflammatory cytokines originating from the tumor and from the host’s tissues, as well as from the immune system [6,8,31]. Patients in the cachectic group presented higher levels of CRP, characterizing the systemic inflammation which is typical of cachexia. Chronic inflammation accounts for an increase in catabolism in peripheral tissues [2] and mitochondrial dysfunction in the skeletal muscle [32].

We show for the first time, an increase in the area of intermyofibrillar mitochondria in patients with cancer cachexia. Altered mitochondrial structure and size have been previously demonstrated in the skeletal muscle of animal models of cancer cachexia [10,14]. Moreover, changes in cardiac muscle mitochondrial morphology and sarcomere disintegration were observed, together with an increase in sarcomeric proteins in skeletal and cardiac muscle in C26-bearing mice [33]. In these animals, cardiac mitochondria presented electron-lucent areas and swelling [33]. Increased mitochondrial area has been found in sarcopenia of old animals [34,35]. Mitochondria in vascular smooth muscle of old Sprague–Dawley rats presented increased size and lower movement extent [34]. Larger sarcomeric mitochondria and longer and more branched intermyofibrillar mitochondria were observed in the gastrocnemius of old mice [35]. These aberrant mitochondria may be related to decreased physical function as observed in aging-related sarcopenia [36]. Furthermore, mitochondrial swelling, measured by mitochondrion permeability transition pore (MPTP) kinetics, has been observed in the skeletal muscle of patients with chronic obstructive pulmonary disease, and abnormalities in the activity of the MPTP may be involved in the increased susceptibly of muscle injury, increased reactive oxygen species production and enhanced cytochrome C release observed in these patients [37]. Disruption of mitochondrial morphology is an indicative of increased MPTP kinetics, while prevention of MPTP opening protects cardiomyocytes viability after lethal hypoxia [38]. Therefore, targeting intervention to prevent MPTP opening and mitochondrial swelling may represent a therapeutic approach to preserve muscle mass in cancer cachexia.

Fis1 gene expression was increased in CC and no changes were observed for MFN2, PGC-1α or TFAM. MFN1, MFN2 and optical protein 1 are enrolled in mitochondrial fusion. MFN2 regulates the development of the fusion complex [32,39]. It has been reported that MFN1 gene expression was decreased in animals with cancer cachexia, although no consensus has been established for MFN2 in the same context [40]. The mitochondrial fission process is coordinated by dynamin-related protein 1 (Drp1) and Fis1 protein. Fis1 is an outer mitochondrial membrane integral protein with a single transmembrane domain and a tetratricopeptide repeat domain facing the cytosol [41]. Fis1 seems responsible for recruiting Drp1 to the outer mitochondrial membrane, which develops active fission sites [32,39]. Drp1 is a GTPase cytosolic protein that translocates to the outer mitochondrial membrane and forms a ring that drives the fission process [42]. Mitochondrial fission segregates dysfunctional mitochondria to be depleted by mitophagy. Increased Fis1 protein expression was reported in animal models of cancer cachexia together with mitophagy [43]. Furthermore, overexpression of functional Fis1 in cell lines drove an increase in autophagosomes while a mutant Fis1 (Fis1^k148R^), which was able to increase mitochondrion fission but not cause dysfunction, did not provoke the same increase in autophagy, indicating that dysfunctional rather than fragmented mitochondria is designated to mitophagy [41].

During the progression of Lewis Lung carcinoma, mitochondrial alterations preceded muscle loss in mice [15]. One week after tumor implantation, there was an increase in mitochondrial oxidative stress in the skeletal muscle; degeneration of mitochondrial network was evident in the second week, and loss of mitochondrial function in the third week [15]. Decrease in skeletal muscle mass was only evident in the fourth week, concomitant to increased Fis1 gene expression and protein content. Higher mitochondrial oxidative stress generation occurred from the first to the third week [15]. In accordance to this, Apc^Min/+^ mice, which develop spontaneous intestinal and colon adenomas, showed diminished MFN1, MFN2 and PGC-1α protein expression in advance to the decrease of muscle mitochondrial content [44]. Reduction in muscle mitochondrial content was concomitant with an increase in apoptosis and autophagy-related protein expression. Furthermore, IL-6 inhibition, through administration of IL-6 receptor antibody, and moderate exercise training were able to prevent mitochondrial dysfunctions in Apc^Min/+^ mice, thus providing evidence that increase in mitochondrial biogenesis at the early phase of cachexia might consist of a therapeutic strategy to attenuate catabolic stimulus in the skeletal muscle [44].

Increase in skeletal muscle autophagy has been formerly described in animal models of cachexia [45,46] and in the skeletal muscle [22] and serum [47] obtained from patients with gastrointestinal cancer with cachexia. In the present study, LC3B II protein expression was found to be increased in the skeletal fiber of patients with cancer cachexia, while no differences were observed for p62, showing that autophagy may be one of the driving factors in muscle loss. In a study from Aversa et al. [22] patients with gastrointestinal cancer and cachexia were compared with patients with weight-stable cancer and with healthy volunteers. There was an increase in the skeletal muscle protein content of LC3B II, p62 and beclin 1, a protein that is also able to initiate autophagy when dissociated of Bcl-2. Higher p62 levels were observed in the two cancer-affected groups, when compared to healthy individuals. The authors suggested that accumulation of p62 may reflect an incomplete autophagy flux, due to lysosomal dysfunctions impairing autophagosome clearance [22]. This hypothesis was also supported by studies with animal models of cancer cachexia in which there was an accumulation of p62 in the skeletal muscle fiber [48,49]. Deletion of ATG7 in mice caused intense muscle atrophy, evidencing that a decrease in autophagy flux is detrimental to muscle mass [50]. Aerobic physical exercise was able to ameliorate muscle mass and restore autophagy flux in the C26 colon carcinoma mice [48]. Considering that autophagocytosis normally degrades p62 and that no changes in p62 were observed in the present study, our results corroborate the role of disrupted autophagy as one of the driving factors of muscle wasting in cancer cachexia.

Pettersen et al. [47] evaluated the autophagy flux in the sera of patients in three different cohorts and described that gastrointestinal cancer patients showed a significant positive association to weight loss and serum autophagy markers, while the same was not observed for lung cancer patients with cachexia (although patients with increased autophagy flux were more likely to present weight loss); and no association was found for hematological and breast cancer patients, in whom cachexia is less prevalent [47]. Furthermore, gastrointestinal cancer patients with cachexia presented this positive association in both genders, whereas weight loss and autophagy flux positive correlation were only reported for male patients with lung cancer-associated cachexia [47]. Moreover, patients with esophageal cancer showed an increase in autophagy-related proteins in the vastus lateralis muscle [24]. This pathway was the main activated proteolytic pathway, as ubiquitin-proteasome, caspase and calpain pathways did not change [24]. Lung cancer-associated cachexia was similarly linked to activation of autophagy lysosomal pathway in the skeletal muscle, as an increase in LC3B II in the quadriceps muscle was found, when compared to pre-cachectic lung cancer patients and healthy control subjects [51].

Additionally, mitochondrial permeability transition (MPT) seems to be involved in autophagy and apoptosis, as MPT pores opening leads to mitochondrial depolarization and swelling [52,53]. Depolarized mitochondria are encompassed by autophagosomes and autolysosomes [54]. Mitochondrial swelling causes mitochondrial outer membrane rupture and release of inner mitochondrial proteins, such as cytochrome C, which, when in the cytosol, triggers apoptosis through caspase 9 activation [55,56]. In the present study, cachexia was related with disruption of mitochondrial morphology and increased autophagy-related protein expression in the skeletal muscle. However, no changes were observed in the mtDNA copy number, indicating that mitophagy was not significantly increased in CC, compared to WSC patients. As suggested by Brown et al. [15], mitochondrial degeneration occurs before muscle wasting, while a decrease in mitochondrial content may be seen in the final stages of cancer-associated cachexia [15] when it may be too late to generate a new pool of functional mitochondria. Mitochondrial fission is mainly responsible for the appearance of depolarized mitochondria, as fission events likely generate a mitochondrion with high mitochondrial membrane potential and a depolarized mitochondrion, which is prone to undergo mitophagy [57]. Twig et al. [57] demonstrated that autophagocytosed mitochondria show three main features: Reduced mitochondrial membrane potential, decreased OPA1, and reduced size. These depolarized mitochondria present a decreased fusion capacity and lower odds of being recruited by polarized mitochondrion to fuse [57].

In addition to aberrant mitochondria and increased autophagy induction in the absence of decreased mitochondrial content, we found higher activation of apoptosis in the skeletal muscle of patients with cancer-associated cachexia. Activated caspase 8 and 9 and phosphorylated p53 (Ser46) protein content were higher in CC, compared to the WSC group, indicating that apoptosis may be also involved in human cachexia-related skeletal muscle loss. Furthermore, apoptotic bodies were present only in the CC group. Caspases are enzymes responsible for cleaving peptide bonds following aspartate residues. Caspase 8 presents *tandem* repeats known as ‘death effector domains’, which are activated following cytokine binding to tumor necrosis factor receptor 1, Fas/APO receptor and associated death receptors. Caspase 9 presents a caspase recruitment domain (CARD) [58]. Cytochrome C, together with dATP, bind to apoptotic protease activating factor-1 and, this last one binds to the CARD domain, activating caspase 9, thereby initiating the proteolytic cascade [28,59]. The extent of mitochondrial damage can regulate cell fate. Mitochondrial outer membrane permeabilization causes the release of cytochrome C from the mitochondrial intermembrane space, which contributes to caspase activation. The overexpression of fusion proteins or silencing Fis1 or Drp1 could reduce cell death and the release of cytochrome C [56].

Increased content of activated caspases 1, 3, 6, 8 and 9 and cytochrome C into the cytosol of the gastrocnemius muscle from cachectic mice bearing the MAC16 tumor has been reported [28]. As mentioned before, mitochondrial swelling leads to mitochondrial membrane rupture and consequently release of cytochrome C followed by caspase 9 activation. In an experiment with cell-free extracts, addition of cytochrome C triggered caspase 9 activation, which was responsible for propagating the cell death signal through activation of caspases 2, 3, 6, 7, 8 and 10 [60]. Furthermore, cachexia was associated with increased apoptosis in the gastrocnemius of Apc^Min,−/+^ mice [27] and in rats bearing the Yoshida AH-130 ascites hepatoma [61]. Patients with gastrointestinal cancer and cachexia showed higher DNA fragmentation and increased cleavage of the cellular membrane protein anti-poly (ADP-ribose) polymerase in the skeletal muscle, when compared with healthy subjects, suggesting augmented apoptotic process [17]. Moreover, animal models of cachexia show enhanced autophagy, concomitant with apoptosis in skeletal muscle [62,63,64].

There are several interconnected signals between autophagy and apoptosis. Bcl-2 inhibits autophagy and apoptosis through its interaction with Beclin-1 and pro-apoptotic proteins, respectively [65]. JNK can phosphorylate Bcl-2 at Thr69, Ser70 and Ser87, decreasing its interaction with Beclin-1 and pro-apoptotic proteins, which could initiate autophagy and apoptosis, respectively. Akt is able to inhibit autophagy and apoptosis by phosphorylating Beclin-1 and BAD [65]. Moreover, mammalian target of rapamycin (mTOR) is induced by Akt and can repress ULK1 complex inhibiting one of the initial steps of autophagy [65]. The localization of p53 influences its action. When in the cytoplasm, p53 inhibits autophagy by blocking ULK1 complex [65,66]. Phosphorylation of p53 drives its translocation from the cytosol to the nucleus, where it initiates the transcription of several genes related to adaptation to stress, arrest of cell cycle, autophagy and/or apoptosis [65]. Under stress, p53 can also translocate to the mitochondrial matrix and stimulate the permeability transition pore opening [65,67].

In opposition to the effects of cachexia in skeletal muscle, physical exercise is able to increase mitochondrial content and improve mitochondrial function [68]. Apc^Min/+^ mice overexpressing IL-6 showed increased muscle wasting with dysfunctional mitochondria [44]. Exercise training prevented the increase of ATG5, Beclin 1 and LC3b protein expression, as well as BAX mRNA expression in the skeletal muscle of Apc^Min/+^ mice [44], indicating physical exercise to modulate mitochondrial stability, autophagy and apoptosis in the skeletal muscle, hence preventing the cachexia-induced deleterious alterations [44]. In another animal model of cancer-associated cachexia, the C26 colon carcinoma mice, spontaneous aerobic exercise diminished autophagy and restored the autophagic flux in the skeletal fiber, in addition to preventing skeletal muscle loss [48]. Muscle loss can be worsened during chemotherapy, in which mitochondrial content is decreased and mitophagy increased, as reported for C26 mice treated with oxaliplatin and 5-fluorouracil (oxfu) [69]. Physical exercise was able to prevent muscle loss and increased mitophagy in the skeletal muscle of tumor-bearing mice treated with oxfu; however, in the last stages of cancer cachexia, treadmill running worsened survival of these animals, indicating that exercise may have an optimal time to be performed within the chemotherapy course [69]. The positive effects of physical exercise may prevent mitochondrial disruption and preserve muscle mass in humans as well [70,71].

It is important to consider the limitations of the present study. The small sample number may not represent the entire population of patients with cancer. In addition, measurements were not always performed with the same sample number, as muscle biopsies generally provided a limited amount of tissue. Low levels of hemogloblin have been positively associated with low muscle mass in different cohorts of elderly subjects [72,73]. Although hemoglobin levels of CC patients indicating mild anemia, hypoxia [38] and iron deficiency [74] have been related to mitochondrial alterations and therefore, should not be ignored. The increase in autophagy-related proteins does not directly indicate an increase in autophagic flux. Furthermore, sexual dimorphism plays an effect in skeletal muscle affected by cachexia: Although men have greater muscle mass than women, weight loss and loss of muscle mass are greater in male than female cancer patients [75,76]. A shortcoming of this study is that the number of female patients was lower than that of male patients. Nevertheless, we were able to show enhanced content of proteins related to apoptosis in the muscle of cachectic patients (both male and female), indicating that this pathway is very likely activated in the skeletal muscle, albeit not providing a proof of concept. To the best of our knowledge this is the first report of these findings in human cancer cachexia.

## 4. Materials and Methods

### 4.1. Patients Recruitment

Patients with gastric and colorectal cancer were recruited after signature of the fully informed consent form. All proceedings were performed following the Declaration of Helsinki and approved by the Ethics Committee on Research Involving Human Subjects of the Institute of Biomedical Sciences/University of São Paulo and by the Human Ethics Committee of the University Hospital/University of São Paulo (CAAE nº 62640216.2.00005467; 18116213.2.3002.5479; 00475118.7.0000.5467). Rectus abdominis muscle biopsies were collected during the surgery for tumor excision. Patients were further divided into Weight-Stable Cancer (WSC, *n* = 20) and Cachectic Cancer (CC, *n* = 24) groups. Supplemental Tables S1 and S2 presents what analyses were performed in each patient. Cancer-associated cachexia was diagnosed following the criteria proposed by Evans at al. (2008) [1], in addition to the screening of plasma C-reactive protein (CRP), as in the Glasgow Prognostic Score [77] and with the results from the following questionnaires (to assess the presence of symptoms related to cachexia): EORTC QLQ-STO22 [78], CASCO [79] and Anorexia Score (FAACT-ESPEN) [80]. Anthropometric data (changes in body weight, current body weight and height) were collected at hospital admission. The inclusion criteria were the following: Female and male patients (35–85 years old) undergoing gastrointestinal tract surgery for tumor excision were selected in partnership with surgeons from the University Hospital of the University of São Paulo (HU-USP) or of the Santa Casa de Misericordia Hospital. All patients selected for the study were submitted to surgery. The exclusion criteria encompassed the following: Enrolled patients were not receiving chemotherapy, radiotherapy, opioids, or continuous anti-inflammatory treatment. Patients presenting chronic inflammation from other etiologies apart from cachexia, as well as those with auto-immune disorders and/or patients with BMI > 29.9 Kg/m^2^ were not engaged in the study.

### 4.2. Blood and Serum Analyses

Blood was collected prior to the surgical procedure—on the day of hospitalization or immediately previous to anesthesia during the surgical procedure—by a trained health professional, placed in tubes and then centrifuged at 3000 rpm for 15 min at 4 °C to obtain serum and plasma, which were transferred to plastic microtubes and stored at −80 °C for posterior analyses. Serum CRP (ultrasensitive CRP Turbiquest plus, cat. nº 331, Labtest, Lagoa Santa, MG, Brazil), albumin (cat. nº 19, Labtest, Lagoa Santa, MG, Brazil), glucose (cat. nº 133, Labtest, Lagoa Santa, MG, Brazil), triacylglycerol (cat. nº 87, Labtest, Lagoa Santa, MG, Brazil), total cholesterol (cat. nº 76, Labtest, Lagoa Santa, MG, Brazil), LDL cholesterol (cat. nº 129, Labtest Lagoa Santa, MG, Brazil) and HDL cholesterol (cat. nº 98, Labtest, Lagoa Santa, MG, Brazil) concentration was assessed with colorimetric commercial kits in an automatic analyzer with high performance for biochemical and turbidimetric tests (LABMAX 240^®^ equipment, Labtest, Lagoa Santa, MG, Brazil). Hemoglobin values were acquired from the patients’ hospital records, previous to surgery.

### 4.3. Transmission Electron Microscopy and Mitochondrial Area

For the ultrastructural studies, 2 mm of the muscles were post-fixed with 1% paraformaldehyde, 2.5% glutaraldehyde, 2.5 mM CaCl2 solution in 0.1 M Sodium Cacodylate buffer (pH 7.2–7.4), and prepared for electron microscopy analysis. After inclusion in Spurr’s kit (cat#14300, Electron Microscopy Sciences, Hatfield, PA, USA), the 250-nm semi-thin sections were obtained with an ultra-microtome (Leica EM UC6, LEICA, St Gallen, Switzerland). Ultra-thin sections (70 nm) were collected onto copper grids (200-mesh) and contrasted with 2% Uranyl Acetate and Lead Citrate. The samples were observed with a FEI TECNAI G20 (FEI Company, Eindhoven, The Netherlands), with a resolution of 4000 × 4000 pixel. Intermyofibrillar mitochondria area was calculated, employing the ImageJ Software (open-source, image processing program, Bethesda, Maryland, MD, USA); after 10× magnification serial photographs acquisition, 8–12 images of each of the samples from each of the 2 groups, were analyzed.

### 4.4. Gene Expression

Muscle mRNA was extracted using TRIzol^®^ (Trizol reagent—Invitrogen, Life Technologies, Carlsbad, CA, USA) following the manufacturer’s instructions. cDNA was synthetized with a commercial kit (High Capacity cDNA Reverse Transcription Kit, Life Technologies). Fast SYBR green master mix (Fast SYBR^®^ Green Master Mix, Thermo Fisher Scientific, Vilnius, Lithuania) was used to perform real-time qPCR, in a QuantStudio 12K Flex Rea l-Time PCR System instrument (Applied Biosystems, Carlsbad, CA, USA) and specific primers for each gene (Invitrogen, Life Technologies, Carlsbad, CA, USA). Relative mRNA expression was standardized to the endogenous housekeeping gene gyceraldehyde-3-phosphate dehydrogenase (GAPDH) and calculated employing the ΔΔC_T_ method. No differences between groups were observed for the GAPDH gene expression. The sequence of the sense (forward—F) and antisense (reverse—R) primers used for amplification were: Fission protein 1 (Fis1) F—CGGAGCAAGTACAATGATGAC, (Fis1) R—CCAGGTAGAAGACGTAATCCC; Mitofusin 2 (MFN2) F—ATGCATCCCCACTTAAGCAC, (MFN2) R—CCAGAGGGCAGAACTTTGTC; mitochondrial transcription factor (TFAM) F—AAGATTCCAAGAAGCTAAGGGTG, TFAM R—CGAGTTTCGTCCTCTTTAGCA; peroxisome proliferator-activated receptor gamma coactivator 1 alpha (PGC-1α) F —TCAAGCCACTACAGACACC, PGC-1α R—TCTCTGCGATATTCTTCCCT; GAPDH F—CCTCTGACTTCAACAGCGAC, GAPDH R—CGTTGTCATACCAGGAAATGAG.

### 4.5. Skeletal Muscle Protein Quantification and Western Blot Analysis

Total protein extraction was carried out using about 50 mg of frozen skeletal muscle tissue, which was homogenized on ice with a Polytron in radioimmunoprecipitation assay (RIPA) buffer (10 mM Tris base, 0.01 mM EDTA, 0.1 mM Sodium Chloride and 1% Triton X-100), with proteinase and phosphatase inhibitors (Roche^®^, Mannheim, Germany); and centrifuged at 14,000× *g*, for 30 min, at 4 °C. The upper phase was collected and placed in a new tube and centrifuged at 14,000× *g*, for 30 min, at 4 °C. Total protein amount from the upper phase of the second centrifugation was quantified employing Bradford protein assay (Bio-rad Laboratories; Hercules, CA, USA), against a bovine serum albumin standard curve, and 40 µg of proteins containing Laemmli sample buffer were separated using precast gels (Bolt Bis-Tris Plus gels 4–12%, Thermo Fisher Scientific, Carlsbad, CA, USA). Proteins were transferred from gels to nitrocellulose membranes (Amersham Protran^®^, GE Healthcare, Germany) at 80 V for 60 min (Trans-Blot Turbo Blotting System, Bio-Rad, Hercules, CA, USA) in transfer buffer (20mM Tris, 150mM Glycine, and 20% Methanol). Membranes were blocked in 5% bovine serum albumin in TBS-T (0.1% Tween 20) for 3 h and then incubated overnight at 4 °C with primary antibodies against microtubule-associated protein 1 light chain 3B (LC3B) (#2775, rabbit, Cell Signaling, Danvers, MA, USA), p62/SQSTM1 (p62), (#5114, rabbit, Cell Signaling, Danvers, MA, USA), autophagy protein (ATG) 5 (#2630, rabbit, Cell Signaling, Danvers, MA, USA), ATG7 (#2631, rabbit Cell Signaling, Danvers, MA, USA) and GAPDH (Santa Cruz Biotechnology, sc-25778, rabbit, Dallas, TX, USA). Next, membranes were washed for 10 min, 3 times with TBS-T, followed by incubation with anti-rabbit IgG, HRP-linked antibody (Cell Signaling, #7074S) for 2 h and then, washed again with TBT-T for 10 min, 3 times. Protein bands were detected with HRP substrate (Luminata Immobilon Forte Western HRP substrate, Merck, Darmstadt, Germany) in a gel documentation system (SyngeneG:boxChemi|Gel Documentation and ECL Detection, Syngene, Frederick, MD, USA). The optical density of the antigen-antibody complex was quantified as pixels employing the Image J Analysis Software (http://rsb.info.nih.gov/ij/). GAPDH was adopted as control protein standard.

### 4.6. Apoptosis-Related Proteins Quantification

Apoptosis-related proteins, Akt (pS473), Bcl-2 (Ser70), Bcl-2-associated agonist of cell death (BAD) (Ser112), p53 (Ser46), JUN N-terminal kinase (JNK) (Thr183/Tyr185), caspase 8 (Asp384) and caspase 9 (Asp315) were quantified in 25 µg of protein from skeletal muscle homogenate with a commercial kit (Car. nº 48-669MAG, Merck-Millipore, St. Charles, MO, USA), employing Multiplex technology in a Magpix^®^ instrument (Life Technologies, Grand Island, NY, USA).

### 4.7. Mitochondrial DNA Copy Number

To compare the levels of mitochondrial DNA (mtDNA) to nuclear DNA, 25 mg of skeletal muscle tissue were disrupted using a digestion buffer (PureLink^®^ Genomic Digestion Buffer, Thermo Fisher Scientific) with proteinase K. Total DNA was extracted using phenol-chloroform-isoamyl alcohol (25:24:1, Sigma-Aldrich ref.P3803, Steinhein, Germany). DNA quantity and quality were accessed with a spectrophotometer (Synergy H1, BioTek, Winooski, VT, USA). A total amount of 3 ng/µL was used to perform real time qPCR as described by Rooney et al. (2015) [81] with Fast Sybr Green master mix (Fast SYBR^®^ Green Master Mix, Thermo Fisher Scientific, Vilnius, Lithuania USA). The amplification primers used were the following: Mitochondrial—tRNA-Leu (UUR) *F*—CACCCAAGAACAGGGTTTGT; *R*—TGGCCATGGGTATGTTGTTA; nuclear—B2-microglobulin *F*—TGCTGTCTCCATGTTTGATGTATCT; *R*—TCTCTGCTCCCCACCTCTAAGT. The average Ct values from duplicate reaction of tRNA-Leu (UUR) amplification were subtracted from the B2-microglobulin Ct to obtain ΔCt. Relative mtDNA content was calculated using the following equation: mtDNA relative copy number = 2 × 2^ΔCT^.

### 4.8. Statistical Analysis

All parameters were firstly submitted to a normality test. Preliminary analysis was carried out to ensure that the assumptions of normality or homoscedasticity were not violated. When normal distribution was observed, Student’s *t*-test was used to compare means between groups and data are expressed as means and standard errors. The Mann–Whitney test with multiple comparisons was employed for non-parametric data, while they are expressed as median and first quartile and third quartile. Statistical significance was set at a *p* value < 0.05 (two tailed). GraphPad Prisma 6 was used to perform statistical analysis.

## 5. Conclusions

This study presents the new finding that intermyofibrillar mitochondria structure is disrupted with an increased area in the skeletal muscle of patients with cancer-associated cachexia, without a decrease in mitochondrial content. Dysfunctional mitochondria may be in the core of skeletal muscle loss in cancer cachexia. Whether mitochondrial alterations are cause or effect of muscle wasting still needs further elucidation. Moreover, we showed that cancer cachectic patients have, concomitantly to increased systemic inflammation, diminished hemoglobin levels, disrupted autophagy and enhanced apoptosis-related protein content, when compared to weigh-stable cancer patients. These results demonstrate that it is more likely that various pathways contribute to skeletal muscle wasting in human cancer cachexia.

## Figures and Tables

**Table 1:** Characteristics and circulating biochemical parameters of WSC and CC patients.

Parameter	WSC	*n*	CC	*n*	*p* Value
Subjects	20		24		
Gender (M/F)	8/12		16/8		
Tumor location (stomach/colorectal)	6/14		8/16		
Tumor stage					
I–II	9		4		
III–IV	8		13		
Age (years)	61.00 ± 2.35	20	61.42 ± 2.68	24	0.90
Previous weight	67.50 ± 2.47	20	73.55 ± 3.40	24	0.17
Weight (kg)	65.90 ± 2.79	20	60.20 ± 2.43	24	0.13
Height (m)	1.64 [1.56; 1.70]	20	1.70 [1.64; 1.72]	24	0.14
BMI (kg/m^2^)	24.69 ± 0.77	20	21.55 ± 0.66	24	**0.003**
Weight loss (%)	0.0 [0.0; 0.0]	20	15.12 [8.84; 23.01]	24	**<0.0001**
Hemoglobin (g/dL)	13.10 [12.20; 14.30]	14	11.30 [9.80; 12.00]	23	**0.0001**
Serum C reactive protein (CRP) (mg/L)	5.07 ± 1.29	17	9.12 ± 0.91	22	**0.01**
Serum albumin (mg/dL)	3.56 ± 0.20	17	3.25 ± 0.19	23	0.28
CRP/albumin ratio	0.18 [0.03; 0.32]	17	0.33 [0.15; 0.42]	22	**0.009**
Serum glucose (mmol/L)	5.23 [4.59; 6.20]	17	5.59 [4.75; 6.03]	22	0.67
Serum triacylglycerol (mmol/L)	1.22 [0.85; 1.75]	17	0.97 [0.81; 1.79]	22	0.60
Serum total cholesterol (mmol/L)	4.42 [3.54; 5.27]	16	3.41 [2.78; 4.61]	22	0.055
Serum LDL cholesterol (mmol/L)	2.96 [1.55; 3.41]	17	1.72 [1.29; 2.24]	21	0.11
Serum HDL cholesterol (mmol/L)	1.04 [0.68; 1.20]	17	0.85 [0.73; 0.98]	22	0.26

Data are expressed as mean ± standard error or as median (first quartile; third quartile). Student’s *t* test was used to compare means and Mann–Whitney test was used to compare median values between WSC and CC patients. WSC—weight-stable cancer patients; CC—cachectic cancer patients. Bold: *p* value lower than 0.05.

**Table 2:** Skeletal muscle gene expression of mitochondrial regulation genes in WSC and CC.

Gene	WSC	CC	*p* Value
Fis1	1.06 ± 0.10	1.50 ± 0.15	**0.03**
MFN2	0.93 [0.71; 1.31]	1.17 [0.84; 1.86]	0.20
TFAM	1.06 [0.78; 1.66]	1.28 [0.93; 1.71]	0.38
PGC-1α	0.97 [0.72; 1.12]	0.87 [0.57; 1.14]	0.34

Data are expressed as mean ± standard error or as median [first quartile; third quartile]. RT-PCR was used to assess mRNA expression levels (WSC, *n* = 12; CC, *n* = 16). Student’s *t* test was adopted to compare means and Mann–Whitney test was used to compare median values between WSC and CC patients. WSC—weight-stable cancer patients; CC—cachectic cancer patients. Bold: *p* value lower than 0.05.

## Supplementary Materials

The following are available online at https://www.mdpi.com/2072-6694/11/9/1264/s1, Figure S1: Higher magnification of skeletal muscle photomicrograph showing intermyofibrillar mitochondria of WSC and CC, Figure S2. Skeletal muscle nuclei photomicrograph of WSC (*n* = 3) and CC (*n* = 4). Black arrows indicate apoptotic body; white arrow indicate chromatin condensation in CC patients. WSC—weight-stable cancer patients; CC—cachectic cancer patients, Table S1: Analyses performed in each patient of the Weight–Stable Cancer group (WSC), Table S2: Analyses performed in each patient of Cachectic Cancer Group (CC).
