# Supplementary material for: Human Cachexia Induces Changes in Mitochondria, Autophagy and Apoptosis in the Skeletal Muscle
Source: Cancers (Basel). 2019 Aug 28;11(9):1264. doi: 10.3390/cancers11091264 (PMC6770124; doi:10.3390/cancers11091264)
Supplement: Supplementary file 1 [file cancers-11-01264-s001.pdf]

Supplementary Materials: Human Cachexia Induces Changes in Mitochondria, Autophagy and Apoptosis in the Skeletal Muscle

Gabriela S. de Castro, Estefania Simões, Joanna D. Lima, Milene Ortiz-Silva, William T. Festuccia, Flávio Tokeshi, Paulo S. Alcantara, José P. Otoch, Dario Coletti and Marilia Seelaender

Table S1. Analyses performed in each patient of the Weight-Stable Cancer group (WSC).

	Haemoglobin	Serum C reactive protein	Serum albumin	Serum glucose	Serum triacylglycerol	Serum total cholesterol	Serum LDL cholesterol	Serum HDL cholesterol	Mitochondrial area	Fis1	MFN2	TFAM	PGC-1α	Mitochondrial copy number	LC3I	LC3II	p62	ATG7	ATG5	JNK	AKT	BCL2	p53	Caspase 8	Caspase 9
1 WSC	x	x	x	x	x	x	x	x		x	x	x	x	x						x					
2 WSC	x	x	x	x	x	x	x	x		x	x	x	x		x	x	x	x	x						
3 WSC	x	x	x	x	x	x	x	x		x	x	x	x		x	x	x	x	x	x	x				
4 WSC	x	x	x	x	x	x	x	x		x	x	x	x	x						x	x	x	x	x	x
5 WSC	x	x	x	x	x	x	x	x						x											
6 WSC	x	x	x	x	x	x	x	x						x	x	x	x								
7 WSC		x	x	x	x	x	x	x		x	x	x	x												
8 WSC	x	x	x	x	x	x	x	x		x	x	x	x		x	x	x	x	x	x	x	x	x		x
9 WSC	x	x	x	x	x	x	x	x		x	x	x	x	x	x	x	x								
10 WSC	x	x	x	x	x	x	x	x		x	x	x	x	x						x	x	x	x	x	x
11 WSC	x	x	x	x	x	x	x	x		x	x	x	x	x											
12 WSC	x	x	x	x	x	x	x	x		x	x	x	x												
13 WSC	x	x	x	x	x	x	x	x						x	x	x	x	x	x						
14 WSC	x	x	x	x	x	x	x	x		x	x	x	x												
15 WSC		x	x	x	x	x	x	x						x											
16 WSC	x								x						x	x	x	x	x	x	x	x	x	x	x
17 WSC	x	x	x	x	x	x	x	x		x	x	x	x	x											
18 WSC		x	x	x	x		x	x	x						x	x	x	x	x	x	x	x	x		x
19 WSC									x					x						x	x	x	x	x	x
20 WSC														x						x	x	x	x	x	x

Table S2. Analyses performed in each patient of Cachectic Cancer Group (CC).

	**Haemoglobin**	**Serum C reactive**	**Serum albumin**	**Serum glucose**	**Serum triacylglyc**	**Serum total**	**Serum LDL**	**Serum HDL**	**Mitochondrial area**	**Fis1**	**MFN2**	**TFAM**	**PGC-1α**	**Mitochondrial copy**	**LC3II**	**LC3I**	**p62**	**ATG7**	**ATG5**	**JNK**	**AKT**	**BCL2**	**p53**	**Caspase 8**	**Caspase 9**
1 CC	x	x	x	x	x	x	x	x		x	x	x	x							x	x	x	x	x	x
2 CC	x	x	x	x	x	x	x	x		x	x	x	x							x	x	x	x	x	x
3 CC	x	x	x	x	x	x	x	x		x	x	x	x		x	x	x	x	x						
4 CC	x	x	x	x	x	x	x	x		x	x	x	x	x											
5 CC	x	x	x	x	x	x	x	x		x	x	x	x	x	x	x	x	x	x	x	x				
6 CC	x	x	x	x	x	x	x	x		x	x	x	x		x	x	x	x	x						
7 CC		x	x	x	x	x	x	x		x	x	x	x	x	x	x	x	x	x						
8 CC	x	x	x	x	x	x		x		x	x	x	x												
9 CC	x	x	x	x	x	x	x	x							x	x	x	x	x	x	x	x	x	x	x
10 CC	x	x	x	x	x	x	x	x		x	x	x	x	x	x	x	x								
11 CC	x	x	x	x	x	x	x	x		x	x	x	x	x	x	x	x			x	x	x	x	x	x
12 CC	x	x	x	x	x	x	x	x						x						x	x	x	x	x	x
13 CC	x	x	x	x	x	x	x	x							x	x	x								
14 CC	x	x	x	x	x	x	x	x						x	x	x	x			x	x	x	x	x	x
15 CC	x	x	x	x	x	x	x	x		x	x	x	x												
16 CC	x	x	x	x	x	x	x	x		x	x	x	x												
17 CC	x	x	x	x	x	x	x	x		x	x	x	x												
18 CC	x	x	x	x	x	x	x	x						x						x	x	x	x	x	x
19 CC	x	x	x	x	x	x	x	x		x	x	x	x												
20 CC	x	x	x	x	x	x	x	x	x	x	x	x	x	x						x	x	x	x	x	x
21 CC	x	x	x	x	x	x	x	x	x	x	x	x	x	x											
22 CC	x	x	x	x	x	x	x	x						x	x	x	x	x	x	x	x	x	x	x	x
23 CC	x								x					x						x	x	x	x	x	x
24 CC	x		x						x					x						x	x	x	x	x	x

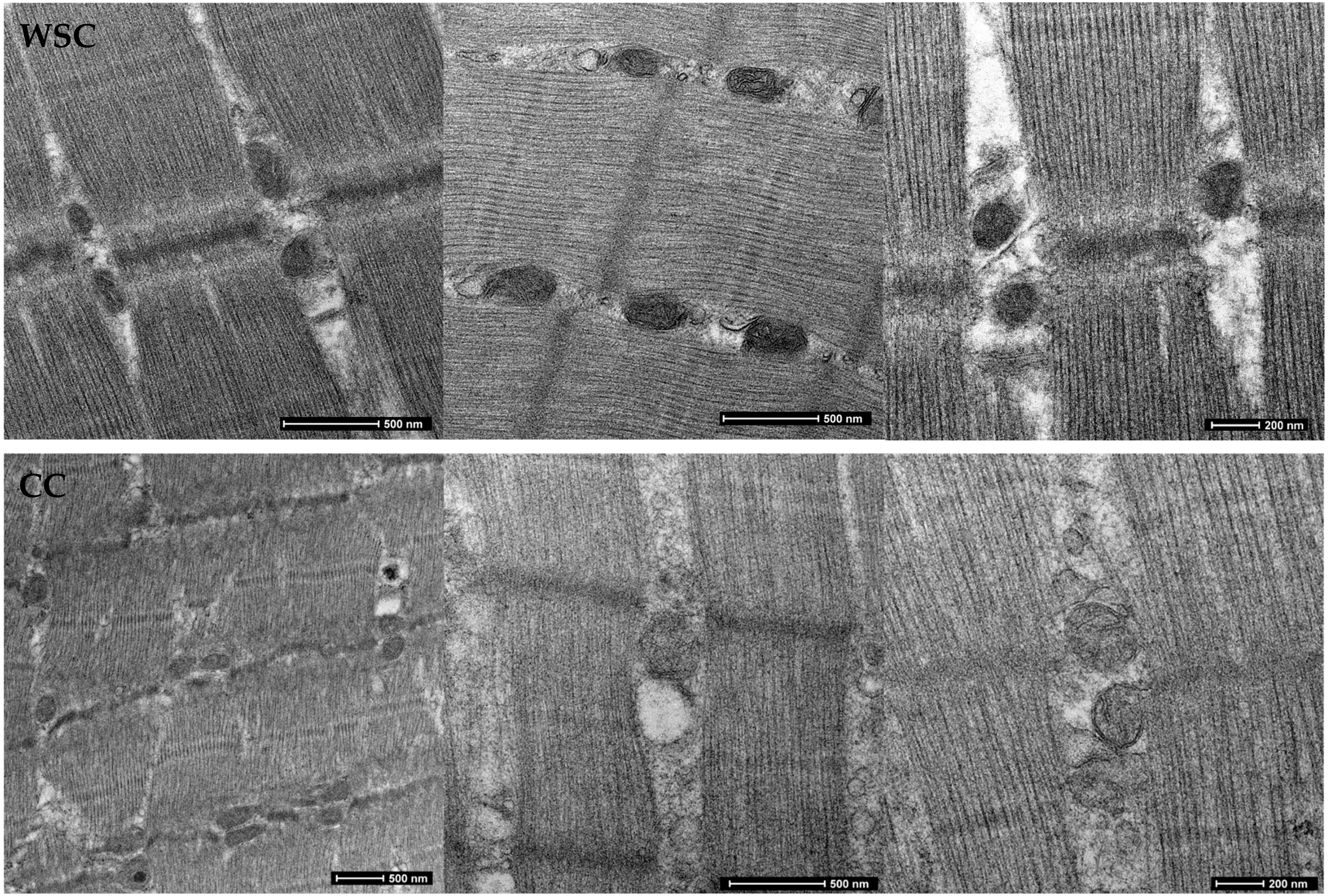

Figure S1. Skeletal muscle photomicrograph showing intermyofibrillar mitochondria of WSC (*n* = 3) and CC (*n* = 3). WSC – weight-stable cancer patients; CC – cachectic cancer.

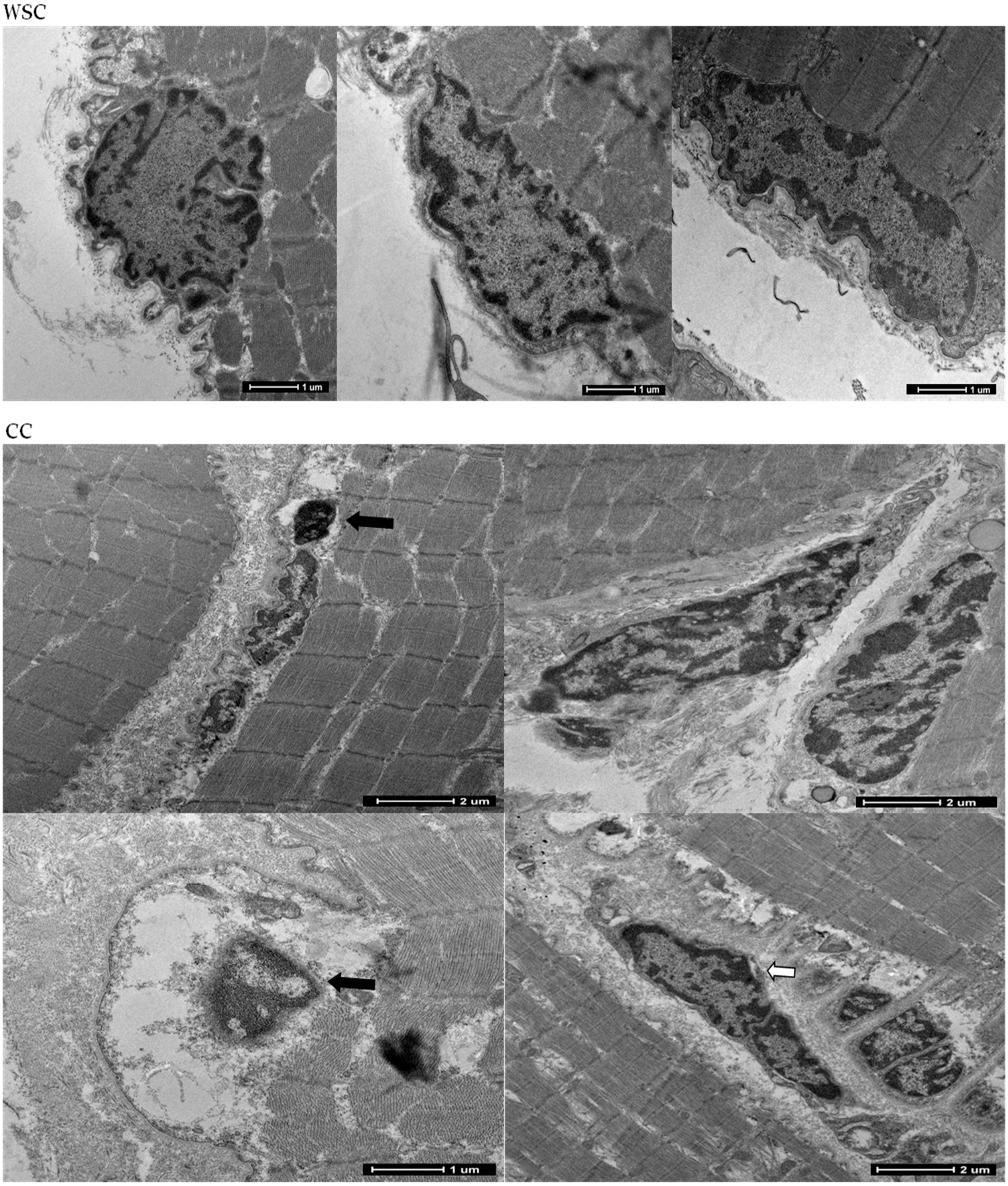

Figure S2. Skeletal muscle nuclei photomicrograph of WSC (n = 3) and CC (n = 4). Black arrows indicate apoptotic body; white arrow indicate chromatin condensation in CC patients. WSC – weight-stable cancer patients; CC – cachectic cancer patients.

© 2019 by the authors. Licensee MDPI, Basel, Switzerland. This article is an open access article distributed under the terms and conditions of the Creative Commons Attribution (CC BY) license (http://creativecommons.org/licenses/by/4.0/).
